# Supplementary material for: High rate of major drug–drug interactions of lopinavir–ritonavir for COVID-19 treatment
Source: Sci Rep. 2020 Dec 1;10:20958. doi: 10.1038/s41598-020-78029-3 (PMC7708981; doi:10.1038/s41598-020-78029-3)
Supplement: Supplementary file 1 — Supplementary Information. [file 41598_2020_78029_MOESM1_ESM.docx]

# High rate of major drug-drug interactions of lopinavir-ritonavir for COVID-19 treatment

**Authors:**

Juan Macías, MD, PhD^1^; Ana Pinilla, RPH^1^; Francisco A. Lao-Dominguez, PharmD^2^; Anaïs Corma, MD^1^; Enrique Contreras-Macias, PharmD^2^; Alejandro González-Serna, PhD^1^; Antonio Gutierrez-Pizarraya, PharmD^2^; Marta Fernández-Fuertes^1^; Ramón Morillo-Verdugo, PharmD^2^; Marta Trigo, MD^1^; Luis M. Real, PhD^1^; Juan A. Pineda, MD, PhD^1^

^1^Infectious Diseases and Microbiology Unit, Hospital Universitario Virgen de Valme. Seville. Spain

^2^Pharmacy, Hospital Universitario Virgen de Valme. Seville. Spain

**Corresponding author**: Juan Macías. Infectious Diseases and Microbiology Unit, Hospital Universitario Virgen de Valme. Avda Bellavista s/n. 41014-Seville. Spain. Email: juan.macias.sanchez@gmail.com

**Original sample size estimation**

We estimated that the frequency of major DDI for LPV-r in hospitalized patients with COVID-19 would be 10%, based on data from HIV clinical trials. To achieve a power of 90%, with a precision of 5%, and assuming a frequency of major DDI of 10%, it is necessary to include 98 subjects treated with LPV-r.

We finally recruited 125 patients with LPV-r. The pre-planned sample size was reached.
